# Zheng’s anchor suturing technique for safe and cosmetic umbilical incision in transumbilical laparoendoscopic single-site surgeries

**DOI:** 10.1007/s00595-022-02585-6

**Published:** 2022-10-15

**Authors:** Yu Chen, Ying Zheng, Liu-Feng Xu, Lin Chen

**Affiliations:** 1 Department of Gynecologic Oncology, West China Second University Hospital, Sichuan University, No. 20, Renmin South Road, Chengdu, 610041 Sichuan People’s Republic of China; 2 Key Laboratory of Birth Defects and Related Diseases of Women and Children (Sichuan University), Ministry of Education, Chengdu, 610041 Sichuan People’s Republic of China

**Keywords:** Incisional complication, Laparoendoscopic single-site surgery, Suture technique

## Abstract

Transumbilical laparoendoscopic single-site surgery (TU-LESS) is a new and evolving surgical method suitable for gynecological diseases, because of its minimal invasion and good cosmetic results. However, since the incision required for this procedure is longer than that for traditional laparoscopy, it may be associated with a higher incidence of postoperative incision complications, such as umbilical hernia, infection, hematoma, and poor wound healing. Moreover, the patient may be left with a misshapen umbilicus because intensive surgery is performed through a single umbilical incision. To minimize the incisional complications and meet patients’ cosmetic expectations, we designed a novel suturing technique, named “Zheng’s anchor suture technique”. This video demonstrates the specific steps and shows photographs of patients’ umbilici that were sutured by this technique, taken after recovery from various operations.

**Supplementary Information:**

The online version of this article (10.1007/s00595-022-02585-6) contains supplementary material, which is available to authorized users.

## Introduction

Transumbilical laparoendoscopic single-site surgery (TU-LESS) is a technique that involves the use of one small skin incision at the base of umbilicus to complete laparoscopic surgical procedures. It can facilitate more convenient specimen extraction, better cosmesis, less postoperative pain, and a shorter recovery period than conventional laparoscopy and laparotomy, so the demand for TU-LESS is increasing [1]. The application of TU-LESS for gynecologic conditions has been expanding rapidly with female patients’ requirements for better cosmesis and with recent advances in surgical equipment, especially for benign diseases.

The umbilicus is an important aesthetic component of the abdomen and its involvement in surgical access influences the cosmetic appearance remarkably. Because intensive procedures are performed through a small incision at the umbilicus in TU-LESS, scar deformation may occur [2]. More importantly, because the approximately 2 cm incision required for TU-LESS is slightly longer than that for traditional laparoscopy, suboptimal suturing may increase the incidence of postoperative incision complications, such as umbilical hernia, infection, hematoma and poor wound healing. To ensure patients’ safety by reducing postoperative complications and reconstructing a symmetric depression of the umbilicus for better cosmesis, we designed a novel suturing technique, named “Zheng’s anchor suturing technique”.

## Methods

**Fig. 1:**
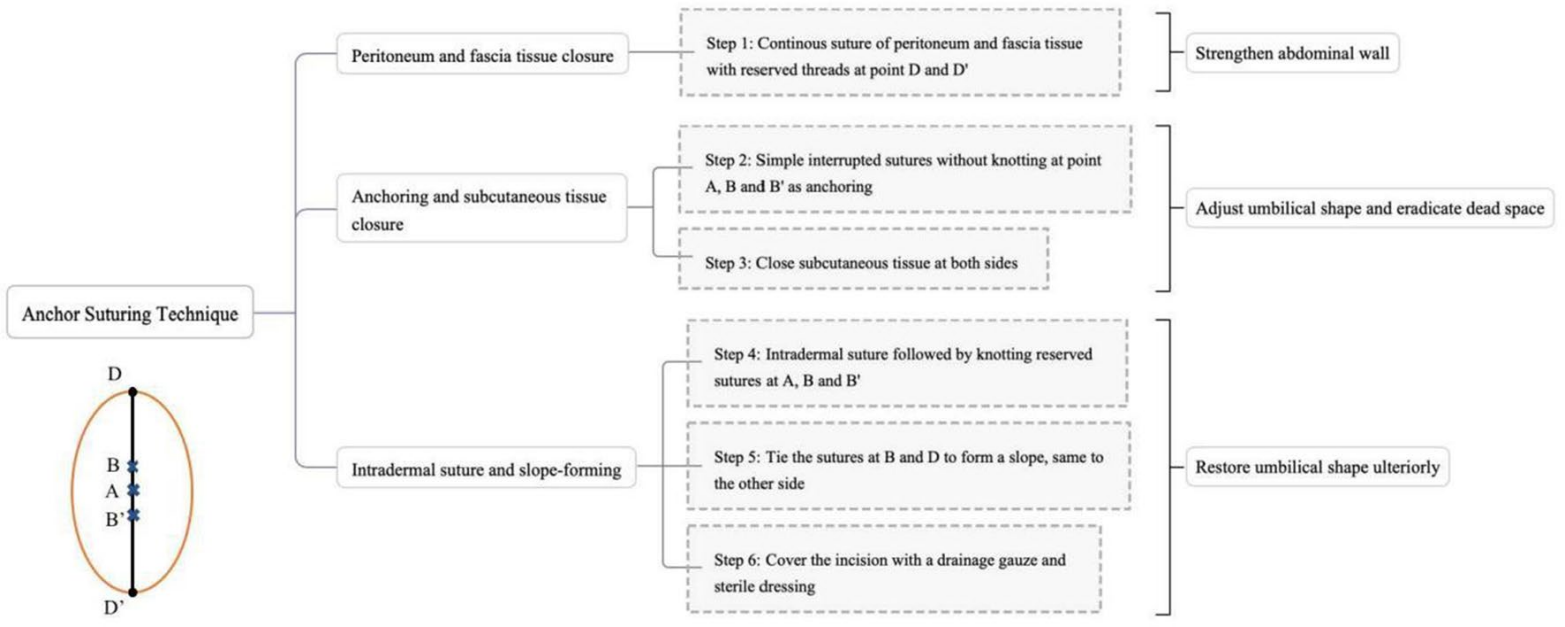
Simple flowchart for Zheng’s anchor suturing technique

On completion of the TU-LESS procedure, the peritoneum and fascia tissue around the umbilical incision are clamped bilaterally with two Allis forceps before removing the port retractor. Thereafter, Zheng’s anchor suturing technique is performed carefully by well-trained surgeons. This technique can be summarized in seven steps as follows (Fig. 1):

Step 1: A continuous suture is placed in the peritoneum and fascia tissue using 2–0 absorbable surgical suture material. After suturing and knotting at both top ends of the incision, the sutures are left and marked as points D and D’.

Step 2 (Anchoring): The middle of the incision is marked as “point A”, which tends to be the most depressed site of a natural umbilicus. About 2 mm from both sides of point A are marked as points B and B’. The fascia and subcutaneous tissue are then closed with simple interrupted sutures (2–0 polyglactin, Vicryl; Ethicon, Inc.) at these three points without knotting.

Step 3: The subcutaneous tissue is closed at point C, between B and D, with simple interrupted sutures or interrupted “figure-of-eight” sutures. The same is repeated on the other side.

Step 4: After completing the continuous intradermal suture using 4–0 absorbable suture material, the subcutaneous tissue sutures are knotted at points A, B, and B’, and trimmed.

Step 5: A slope is formed by tying the sutures at point B and D, and the umbilicus is formed. The sutures at points B’ and D’ are tied in the same way.

Step 6: After a subcutaneous injection of local anesthetic, an appropriative drainage gauze is crumpled and fitted into the depression and the incision is covered with a disposable sterile dressing.

Step 7: The patient can be encouraged to wear an abdominal binder for the initial 2–4 weeks after surgery to promote wound healing.

## Results

Between September, 2017 and April, 2021, a total of 5489 patients underwent TU-LESS with Zheng’s anchor suturing technique in West China Second University Hospital. The entire procedure can be completed in about 30 min for initial exploration by surgeons new to this technique, but this can be reduced to 5–15 min with increasing proficiency. All incisions sutured by this technique are now invisible and the patients expressed great satisfaction with their umbilical appearance a few months after recovering from surgery. Patients were followed up in the clinic or by phone-calls, and 119 incision-related postoperative complications were identified, including infection (*n* = 97, 1.8%), poor wound healing (*n* = 15; 0.27%), umbilical hernia (*n* = 6, 0.11%), and hematoma (*n* = 1, 0.02%). Between July, 2017 and December, 2019, 4 umbilical hernias developed after a total of 2137 TU-LESS procedures, since when an additional 3352 have been performed with only 2 umbilical hernias. The umbilical hernias were diagnosed by ultrasound 1–8 months after initial surgery and two required surgical repair. Among 180 patients diagnosed as obese based on the Chinese criteria of a BMI > 28 kg/m^2^, there were three infections and one incisional disruption, but no umbilical hernias or hematomas. Incisional swelling, exudation, and suppuration were all considered signs of infection and were healed by disinfecting with iodophor solution regularly without the requirement of antibiotic therapy.

## Discussion

Umbilical hernia is a potential problem associated with TU-LESS because of the anatomical weakness of the para-umbilical region, the longer port-site incision, and stretching of the port site for specimen retrieval. All fascial defects greater than 10 mm at the port site should be closed according to current consensus; however, as TU-LESS requires an incision of 2.0 cm or longer [3], the umbilical incision should be closed with a proper suturing technique to prevent incision complications. Umbilical hernia is more likely to develop in patients with a wound infection, obesity, poor nutrition, an immunocompromised state, connective tissue disorders, or postoperative chemotherapy [4]. According to previous studies, the incidence of postoperative umbilical hernia ranges from 0.12 to 13.3% after gynecologic or general surgeries [5]. To reduce the incidence of umbilical hernia after TU-LESS, surgeons have tried various closure techniques and reported satisfactory results [2, 6, 7], but the number of cases in these reports was small and an optimal method to address the different risk factors was not identified.

As a pioneer team conducting TU-LESS procedures in our hospital from 2017, we designed this anchor suturing technique based on the plastic and aesthetic comprehension of the umbilicus, and then shared it with subsequent TU-LESS surgeons and gained consistent affirmation. “Zheng’s anchor suturing technique” was recently recommended in the Chinese expert consensus on the management of umbilical incision in transumbilical laparoendoscopic single-site surgery (2022 edition) [8]. In the present study of 5489 patients who underwent “Zheng’s anchor suturing technique” after TU-LESS procedures there were considerably fewer cases of umbilical hernia than reported in previous studies. The key points of this suturing technique were complete hemostasis, layer-by-layer suturing to strengthen para-umbilical anatomic structures, and restoration of umbilical cosmesis. After the TU-LESS procedure, the port should not be removed immediately. Under the port’s protection of the surrounding tissue, especially the small intestine, it is easy to identify and clamp the peritoneum and fascia layer for continuous suturing. Careful and firm closure of the fascia is essential to prevent umbilical herniation. Moreover, two surgeons’ knots must be secure at both ends to achieve stability as well as better healing. We emphasize the anchoring step, which marks the most impressed point of the umbilicus and restores the natural shape by forming slopes symmetrically on both sides. Tying the sutures at points B and D can eradicate the subcutaneous dead space and prevent postoperative hematoma, which is considered a potential risk factor for surgical site infection. In circumstances like a smaller incision (1.5–2 cm) and deep umbilici, steps 3 and 5 can be omitted. Both ends of the continuous intradermal suture can be unknotted and run subcutaneously for 3–5 mm or turned 1–2 stitches back, maintaining proper tension to knot reserved sutures at points A, B, and B’ with one surgeons’ knot. The cosmetic effects are better in patients with deeper umbilical fossae because the incisions are completely buried inside the umbilici making surgical scars virtually invisible, unless there is scar constitution. Postoperative abdominal distension, coughing, straining on defecation, and other conditions that raise abdomen pressure may lead to incisional disruption. Therefore, the utility of an abdominal binder is recommended for 2–4 weeks to reduce incisional tension, alleviate pain, and promote wound healing. There were six patients with a postoperative umbilical hernia in the present series: two were over 60 years old, two had undergone previous laparoscopic surgery, and five had at least one full-term pregnancy and delivery, which can lead to weakness of the abdominal wall. Since this technique was implemented by different surgeons, including junior residents, we assumed that the incidence of umbilical hernias may ascribe to insufficient closure of the fascia layer, so completion of this technique by or under the guidance of a well-trained surgeon is suggested for safety. Previous studies used literary or picturable descriptions or high-quality illustrations to demonstrate suturing methods for umbilical closure, but these are not intuitive enough for surgical education. Therefore, we made this flash animation (Online Resource 1) to clearly demonstrate the entire procedure of how to make a central umbilical incision and close it with this novel suturing technique. Further comparative studies investigating different suturing techniques for TU-LESS incision are required.

## Conclusion

TU-LESS procedures have the advantages of more minimal invasion and better cosmetic results than multiport laparoscopic surgeries. However, the incidence of incision complications such as umbilical hernias is higher and fascia defects should be reconstructed more securely. Restoration of umbilical cosmesis is also vital for young women undergoing gynecologic procedures. Our anchor suturing technique provides an effective option for TU-LESS umbilical closure to achieve a low rate of incision complications and patient satisfaction of their postoperative umbilical appearance.

## Supplementary Information

Below is the link to the electronic supplementary material.

Supplementary file1 (MPG 50440 KB)

## References

[1] Boruta DM (2016). Laparoendoscopic single-site surgery in gynecologic oncology: An update. Gynecol Oncol.

[2] Menon A, Ganapathi A (2017). Neoumbilicoplasty in a laparoscopic port site: description of a new technique and review of literature. J Clin Diagn Res.

[3] Tonouchi H, Ohmori Y, Kobayashi M (2004). Trocar site hernia. Arch Surg.

[4] Gunderson CC, Knight J, Ybanez-Morano J, Ritter C, Escobar PF, Ibeanu O (2012). The Risk of Umbilical Hernia and Other Complications with Laparoendoscopic Single-Site Surgery. J Minim Invasive Gynecol.

[5] Noh JJ, Kim TH, Kim CJ, Kim TJ (2020). Incisional hernia after 2498 single-port access (SPA) gynecologic surgery over a 10-year period. Sci Rep.

[6] Chang JW, Oh J, Jung US (2018). Umbilical quilting suture technique during single-port laparoscopic surgery. JSLS.

[7] Matsui Y, Satoi S, Hirooka S, Kon M (2015). Simple suturing technique for umbilical dimple wound after single-incision laparoscopic surgery [J]. J Am Coll Surg.

[8] Zheng Y, Xiong GW, Liu J, Sun J, Han L, Shen Y (2022). Micro- and Non-invasive medical single-site and vaginal endoscopy group of Chinese medical doctor association expert consensus on the management of umbilical incision in transumbilical laparoendoscopic single-site surgery (2022 edition). J Pract Obstetr Gynecol.

